# Periodontal Ehlers-Danlos Syndrome Is Caused by Mutations in *C1R* and *C1S*, which Encode Subcomponents C1r and C1s of Complement

**DOI:** 10.1016/j.ajhg.2016.08.019

**Published:** 2016-10-13

**Authors:** Ines Kapferer-Seebacher, Melanie Pepin, Roland Werner, Timothy J. Aitman, Ann Nordgren, Heribert Stoiber, Nicole Thielens, Christine Gaboriaud, Albert Amberger, Anna Schossig, Robert Gruber, Cecilia Giunta, Michael Bamshad, Erik Björck, Christina Chen, David Chitayat, Michael Dorschner, Marcus Schmitt-Egenolf, Christopher J. Hale, David Hanna, Hans Christian Hennies, Irene Heiss-Kisielewsky, Anna Lindstrand, Pernilla Lundberg, Anna L. Mitchell, Deborah A. Nickerson, Eyal Reinstein, Marianne Rohrbach, Nikolaus Romani, Matthias Schmuth, Rachel Silver, Fulya Taylan, Anthony Vandersteen, Jana Vandrovcova, Ruwan Weerakkody, Margaret Yang, F. Michael Pope, Molecular Basis of Periodontal EDS Consortium, Kirk Aleck, Zoltan Banki, Joszef Dudas, Herbert Dumfahrt, Hady Haririan, James K. Hartsfield, Charles N. Kagen, Uschi Lindert, Thomas Meitinger, Wilfried Posch, Christian Pritz, David Ross, Richard J. Schroer, Georg Wick, Robert Wildin, Doris Wilflingseder, Peter H. Byers, Johannes Zschocke

**Affiliations:** 1 Department of Operative and Restorative Dentistry, Medical University of Innsbruck, Innsbruck 6020, Austria; 2 Department of Pathology, Collagen Diagnostic Laboratory, University of Washington, Seattle, WA 98195-7655, USA; 3 Division of Human Genetics, Medical University of Innsbruck, Innsbruck 6020, Austria; 4 MRC Clinical Sciences Centre and Department of Medicine, Imperial College London, London W12 0NN, UK; 5 Institute of Genetics and Molecular Medicine, University of Edinburgh, Edinburgh EH4 2XU, UK; 6 Department of Molecular Medicine and Surgery and Centre for Molecular Medicine, Karolinska Institute, Stockholm 171 76, Sweden; 7 Department of Clinical Genetics, Karolinska University Hospital, Stockholm 171 76, Sweden; 8 Division of Virology, Medical University of Innsbruck, Innsbruck 6020, Austria; 9 Institut de Biologie Structurale (IBS), University Grenoble-Alpes, CEA, CNRS, Grenoble 38044, France; 10 Department of Dermatology, Venereology and Allergology, Medical University of Innsbruck, Innsbruck 6020, Austria; 11 Connective Tissue Unit, Division of Metabolism and Children’s Research Centre (CRC), University Children’s Hospital, Zurich 8032, Switzerland; 12 Department of Pediatrics, University of Washington, Seattle, WA 98195-6320, USA; 13 Department of Genome Sciences, University of Washington, Seattle, WA 98195-5065, USA; 14 Center for Mendelian Genomics, University of Washington, Seattle, WA 98195, USA; 15 Seattle Children’s Research Institute, Seattle, WA 98195-7655, USA; 16 The Prenatal Diagnosis and Medical Genetics Program, Department of Obstetrics and Gynecology, Mount Sinai Hospital, University of Toronto, Toronto, ON M5G 1X5, Canada; 17 Division of Clinical and Metabolic Genetics, Department of Pediatrics, The Hospital for Sick Children, University of Toronto, Toronto, ON M5G 1X8, Canada; 18 Department of Public Health and Clinical Medicine, Dermatology, Umeå University, Umeå 901 87, Sweden; 19 Cologne Center for Genomics, University of Cologne, Cologne 50931, Germany; 20 Department of Biological Sciences, University of Huddersfield, Huddersfield HD1 3DH, UK; 21 Department of Molecular Periodontology, Umeå University, Umeå 901 87, Sweden; 22 Departments of Genetics and Genome Sciences and Pediatrics, Case Western Reserve University Medical Center, Cleveland, OH 44106, USA; 23 Medical Genetics Institute, Meir Medical Center, Kfar Saba 44100, Israel; 24 Maritime Medical Genetics Service, IWK Health Centre, Halifax, NS B3K 6R8, Canada; 25 King’s College London, Department of Medical & Molecular Genetics, Guy’s Hospital, London WC2R 2LS, UK; 26 Department of Surgery and Cancer, Imperial College London, London W12 0NN, UK; 27 West Middlesex University Hospital, Isleworth, Middlesex TW7 6AF, UK; 28 Hospital of St John & St Elizabeth, London NW8 9NH, UK; 29 Department of Medicine (Medical Genetics), University of Washington, Seattle, WA 98195, USA

## Abstract

Periodontal Ehlers-Danlos syndrome (pEDS) is an autosomal-dominant disorder characterized by early-onset periodontitis leading to premature loss of teeth, joint hypermobility, and mild skin findings. A locus was mapped to an approximately 5.8 Mb region at 12p13.1 but no candidate gene was identified. In an international consortium we recruited 19 independent families comprising 107 individuals with pEDS to identify the locus, characterize the clinical details in those with defined genetic causes, and try to understand the physiological basis of the condition. In 17 of these families, we identified heterozygous missense or in-frame insertion/deletion mutations in *C1R* (15 families) or *C1S* (2 families), contiguous genes in the mapped locus that encode subunits C1r and C1s of the first component of the classical complement pathway. These two proteins form a heterotetramer that then combines with six C1q subunits. Pathogenic variants involve the subunit interfaces or inter-domain hinges of C1r and C1s and are associated with intracellular retention and mild endoplasmic reticulum enlargement. Clinical features of affected individuals in these families include rapidly progressing periodontitis with onset in the teens or childhood, a previously unrecognized lack of attached gingiva, pretibial hyperpigmentation, skin and vascular fragility, easy bruising, and variable musculoskeletal symptoms. Our findings open a connection between the inflammatory classical complement pathway and connective tissue homeostasis.

## Introduction

Ehlers-Danlos syndrome (EDS) is a clinically and genetically heterogeneous group of connective tissue disorders defined by joint laxity and skin alterations that include hyperextensibility, atrophic scarring, and bruising.^1^ Periodontal EDS (pEDS, previously EDS VIII), a specific subtype of EDS with autosomal-dominant inheritance, was first identified by Stewart et al. in 1977^2^ and has been subsequently reported in 29 case reports and seven pedigree analyses^3^, ^4^, ^5^, ^6^, ^7^ (MIM: 130080). The defining feature is an EDS phenotype combined with severe periodontal inflammation. In childhood, periodontal inflammation in pEDS is characterized by extensive gingivitis in response to mild plaque accumulation. In the teens, early-onset periodontitis (EOP) leads to inflammatory destruction of dental attachment and premature loss of teeth. Other clinical features previously reported include pretibial hyperpigmentation, acrogeria, skin and gum fragility, scarring, generalized and/or distal joint hypermobility, and bruising out of proportion to trauma. There are single case reports of life-threatening complications like arterial or gastrointestinal ruptures.^8^

In three families, pEDS was previously mapped to a 7 cM (5.8 MB) interval on chromosome 12p13^4^ but so far the genetic cause of the condition has not been identified. We have found that in 17 of 19 families we studied, pEDS is associated with heterozygous mutations in either of two adjacent genes in the linked region: *C1R* (MIM: 613785) (in 15 families) or *C1S* (MIM: 120580) (in 2 families). This identifies a unique link between connective tissue pathology and the classical complement pathway in a monogenic condition.

## Subjects and Methods

### Ethical Considerations

The study was conducted in accordance with the Helsinki Declaration of 1975, as revised in 2000, and was approved as part of the Biobank for Rare Diseases by the Ethics Committee of the Medical University Innsbruck, Austria (study no. UN4501). UK patients were recruited according to Ethics Protocol Reference 11/LO/0883 (West London Research Ethics Committee). US study participants were consented through the University of Washington Research Repository of Heritable Disorders of Bone, Blood Vessels and Skin (IRB protocol 27083) or Cedars-Sinai Medical Center IRB protocols 0359 and 0463. The study was part of the Institution Review Board-approved Repository of Heritable Connective Tissue Disorders at the University of Washington. Each individual or the parents of under-age individuals signed informed written consent before investigation. Consent of individuals was obtained to publish their intraoral photographs.

### Genomic Analysis

Exome-sequence analysis was performed in ten families (families 1, 2, 4, 5, 11, 15–19) by four different groups (Innsbruck, Edinburgh, Seattle Center for Mendelian Genomics, and Seattle Center for Precision Diagnostics), using standard methods. In Innsbruck, the analysis was preceded by linkage studies to define the regions within the previously linked locus that co-segregated with the phenotype. In the others, whole-exome analysis was completed and the analysis performed genome-wide with attention to the region previously identified on chromosome 12. Presence of the same mutation was confirmed in all available affected family members and excluded in the non-affected individuals by Sanger sequencing.

Once we identified two candidate genes, *C1R* and *C1S*, we searched our available laboratory databases for additional families with the possible diagnosis of pEDS and analyzed *C1R* and *C1S* by Sanger sequencing in samples from families 3 and 6–13. Additionally, *C1R* and *C1S* were analyzed by Sanger sequencing in samples from 11 individuals who had been referred for diagnostic testing to exclude vascular EDS (MIM: 130050) and in samples from 71 individuals diagnosed with aggressive periodontitis. Aggressive periodontitis (MIM: 170650) is a main differential diagnosis of pEDS. It is a complex genetic disease and is characterized by a high rate of disease progression, an early age of onset, and the absence of systemic diseases.^9^

### Clinical Investigations

Clinical data were obtained from all mutation-positive families (families 1–17) through detailed questionnaires (available from the authors on request), which were completed with the attending physicians or—if not otherwise possible—by the family members.

In families 1 and 14, the clinical diagnosis of early-onset periodontitis was based on four or more interproximal sites with clinical attachment loss ≥ 6 mm (not on the same tooth) and four or more interproximal sites with probing pocket depth ≥ 5 mm, or history of complete tooth loss due to tooth mobility at an age of ≤35 years. In other individuals the case finding depended on severe periodontal bone loss or tooth loss due to tooth mobility at young ages (<35 years), validated radiographically or by history and recollection. Additional investigations in family 1 included electron microscopic analysis of cultured fibroblasts of skin biopsy samples and collagen biochemical analyses, as well as activity analyses of the classical complement pathway (CH50-assay), using standard methods.^10^, ^11^, ^12^

### Statistical Methods

Standard descriptive methods were used to summarize the clinical parameters studied.

### Variant Modeling

To map the position of identified variants, 3D models of C1r and C1s were constructed using previously determined X-ray structures. The C1s model is a composite structure obtained after superimposing the PDB structures 1ELV and 4LMFA onto 4LOT.^13^ The C1r model combines the X-ray structure of its CCP1-CCP2-SP structure^14^ and a model of the CUB1-EGF-CUB2 interaction domain based on its homology with C1s.^15^ Pymol was used to draw the structural illustrations.^16^

### Expression Studies

*C1R* mutations c.149_150TC>AT (p.Val50Asp), c.927C>G (p.Cys309Trp), and c.1113C>G (p.Cys371Trp), as well as a 26 bp frameshift insertion at position c.899_900 as non-functional control, were generated by site-directed mutagenesis (QuikChange Lightning kit, Agilent Technologies) in a mammalian *C1R* expression vector (GenScript). Vectors were transfected into *C1R*-negative HEK293 cells (Sigma Aldrich). Test for mycoplasma contamination (Minerva Biolabs) in cells was negative. Stably transfected cells were selected in the presence of G418 (600 ng/mL; Sigma Aldrich). Cells were rinsed two times in PBS to remove serum components and incubated in serum-free medium (LONZA Inc.) for 3 days. Thereafter cells and supernatants were harvested separately; supernatants were concentrated to 1/20 volume using centrifugal concentrators (Sartorius). For protein isolation, cells were disrupted using RIPA buffer containing protease inhibitor (SIGMA), and the protein concentration was photometrically determined using Bradford reagent (BIORAD).

Western blot analysis of cell lysates and supernatants was performed with C1r-specific primary antibody diluted 1:1,000 (Abcam cat# ab66751, RRID: AB_1860204; which recognizes the first 100 residues of the A chain) as described.^17^ Normal human serum (diluted 1:10) and non-transfected HEK293 cells were used as controls.

Transfected and non-transfected HEK293 cells were fixed in suspension with Karnovsky’s formaldehyde-glutaraldehyde fixative for 1 hr, followed by rinsing in 0.1 M Cacodylate buffer. All specimens were postfixed in 3% aqueous osmium tetroxide, contrasted with 0.5% veronal-buffered uranyl acetate, embedded in Epon 812 resin. Sections were examined by transmission electron microscopy (Phillips EM 400, FEI Company Electron Optics; operating voltage 80 kV) as described.^18^

## Results

### Genetic Results

**Figure 1:**
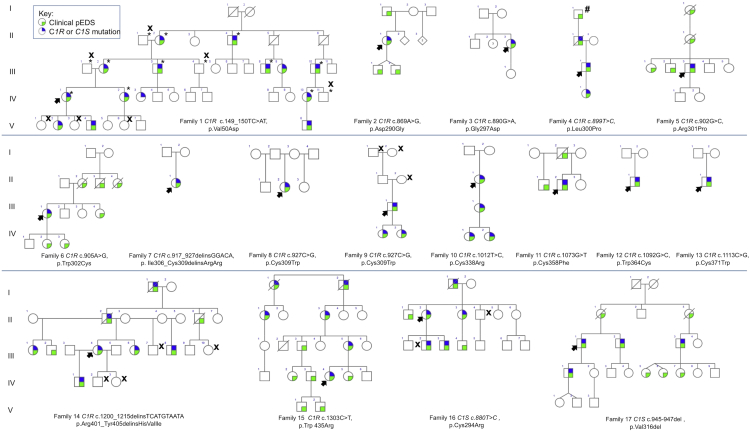
The Pedigrees for 17 Families with *C1S* or *C1R* Mutations The colored symbols are defined in the key. X denotes individuals with normal result of molecular testing; asterisk (^∗^) indicates samples included in linkage studies in family 1. Hatch sign (#) indicates individual in family 4 described as “affected” in a previous publication^3^ but not confirmed by molecular testing. For families 5 (Rahman et al.^4^), 8 (Hartsfield and Kouseff^22^), and 11 (Stewart et al.^2^), a more complete pedigree has been previously published.

A total of 19 families from USA and Europe comprising 107 individuals with the clinical diagnosis of pEDS were available for molecular investigations. Genome-wide linkage analysis in family 1 confirmed the previously reported locus for pEDS on chromosome 12p13.1. Exome sequencing identified sequence variants in *C1R* (GenBank: NM_001733.4) in six families and in *C1S* (GenBank: NM_201442.2) in two families. Subsequent targeted sequencing revealed *C1R* sequence variants in nine additional families (Figure 1, Table 1). None of the identified variants was listed in the ExAC database of more than 60,000 exomes of normal individuals, the 1000 Genomes database, ClinVar, or the SNP data base (last accessed 03/2016).

No potentially pathogenic mutations in *C1R* or *C1S* were identified in families 18 and 19, previously reported to be affected by pEDS but not available for clinical re-assessment,^5^, ^19^ in 11 individuals clinically diagnosed with vascular EDS, or in 71 individuals diagnosed with aggressive periodontitis but without EDS-like features. *C1Q* was sequenced in families 18 and 19, but no potentially pathogenic variants were identified by exome sequencing.

### Protein Variant Modeling

C1r and C1s are multidomain proteins that share similar structures (Figure 3A). C1r and C1s are assembled into a Ca^2+^-dependent C1s-C1r-C1r-C1s tetramer that associates with the recognition protein C1q (Figures 3A–3C).^13^, ^20^ Most of the alterations in C1r and C1s structure involved the domains CUB2 and CCP1 in C1r and the domain CCP1 in C1s (Table 1, Figures 3D and 3E). The C-terminal catalytic serine-protease domains were unaffected. The C1r variants in families 7–11 and 13 affected paired cysteines involved in disulfide bonds that stabilize the C1r CCP1 module. The variant in family 16 substitutes a cysteine in the CCP1 module of C1s. The introduction of an additional cysteine in C1r CUB2 or CCP1 (families 6 and 12) could affect the native disulfide bond formation. The deletion of five residues and insertion of three amino acids in C1r CCP2 in family 14 changes the structure adjacent to a cysteine (position 406) involved in the disulfide bond (406–447) that stabilizes the CCP2 module.

### Expression Studies

To assess the effects of identified variants, we overexpressed mutant C1r (p.Val50Asp, p.Cys309Trp, p.Cys371Trp), wild-type C1r, and a C1r non-functional control as cDNAs in HEK293 cells. Western blot analyses were performed with a monoclonal antibody directed against the N-terminal part of C1r (which includes the binding domains); the antibody recognizes the full-length protein, the A chain generated by C1r activation, and the α-fragment generated by autoproteolysis (Figure 4).^21^ Analysis of cell culture supernatant showed C1r protein only in medium of cells transfected with wild-type *C1R* (the 35 kDa autoproteolytic α-fragment; Figure 4A). Analysis of lysed cells identified an additional band at approximately 55 kDa (A chain) in cell lines transfected with plasmids harboring *C1R* missense mutations; this band was not present in the other cell lines (Figure 4B). Electron microscopy showed an increased proportion of dilated rough endoplasmic reticulum (RER) cisternae in *C1R* mutation-transfected HEK cells compared to wild-type and non-transfected control cells (Figures 4D–4F). Semiquantitative analysis of randomly selected section profiles showed RER dilatation in 36/63 profiles in cells transfected with p.Cys371Trp compared to 18/60 profiles in cells transfected with the wild-type sequence and 13/38 profiles in non-transfected cells.

### Clinical Characteristics of Periodontal EDS

The 17 families with mutations in *C1R* or *C1S* comprised 93 individuals with pEDS (Figure 1). Clinical characteristics in individuals with *C1R* or *C1S* mutations are summarized in Table 2. Family descriptions as well as clinical data of each individual are provided in the Supplemental Data.

**Figure 2:**
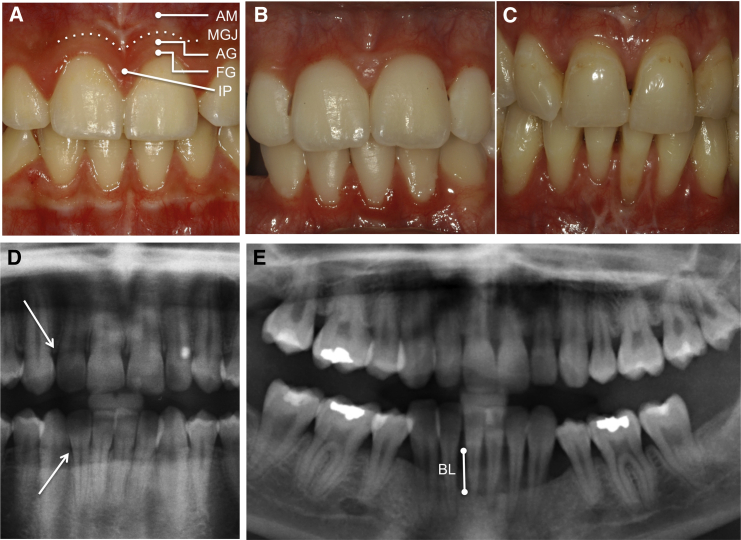
Oral Features of Periodontal EDS (A) Gingival tissues of a non-affected control child (1:V-1). The gingiva is subdivided into the non-attached free gingival margin (FG), the attached gingiva (AG), and the interdental papilla (IP). The gingival epithelium is keratinized and performs a protective function during mastication. The attached gingiva is tightly bound to the periostum via collagen structures. The border between attached gingiva and alveolar mucosa (AM) is the mucogingival junction (MGJ). The oral mucosal epithelium is non-keratinized and only loosely connected to the periostum; therefore, it is more fragile. (B and C) Gingival tissues of an affected child (1:V-2) (B) and of an affected adult (1:IV-1) (C). The attached gingiva is missing; the oral mucosa extends to the free gingival margin and the interdental papillae. (D) Dental radiograph of a non-affected individual (1:IV-4). The alveolar crest is the most cervical rim of the alveolar bone (arrow); in health, it is located approximately 1 mm apical to the cemento-enamel junction (border between dental crown and root). (E) Dental radiograph of an affected individual 1:IV-2, aged 24 years. Notice periodontal bone loss (BL) in the lower jaw. The alveolar crest is now located more apically.

Defining oral features of pEDS are (1) extensive gingival inflammation in response to mild dental plaque accumulation and (2) early-onset periodontitis (EOP) characterized by a rapid destruction of the periodontal attachment apparatus in the teens. EOP was present in 99% of clinically or genetically ascertained adults (which partly reflects EOP as a selection criterion). The median age of the periodontal diagnosis—in some individuals the age of first periodontal tooth loss—was 14 years (range 2–35 years). One affected adult (1:IV-3) did not have periodontitis at the age of 24 years, but had extreme gingival recession. Gingival recession (i.e., receding gums) was diagnosed in 98% of individuals. Affected individuals, when specifically examined (families 1, 4, 8, 11, 14), had a striking lack of attached gingiva causing oral tissue fragility (Figure 2), which was a unique structural gingival anomaly.

Another defining feature of pEDS was pretibial hyperpigmentation (83%). No pretibial changes were found in family 1 where the skin had normal elasticity but appeared rather soft and dry. Almost all affected individuals had easy bruising (96%), skin fragility (83%), and mild skin hyperextensibility (73%). Abnormal scars (atrophic or wide) were present in 50% of individuals. Some individuals had additional dermatological findings such as marked facial flushing, thin nails, or thin hair. One individual (9:II-1) reported difficulties in wound healing, with open wounds that took months or even years to heal. Joint hypermobility was not a consistent finding (44%), and if present was mild and often limited to small joints. Joint pain, scoliosis, and pes planus were rare. Affected individuals in family 4 reported no musculoskeletal symptoms.

Of the affected individuals, 40% were prone to recurrent infections such as otitis media, herpes zoster, bladder infections, empyema, kidney infections, or pneumonia. There was a history of aneurysms in 16% of affected individuals (families 6, 7, and 15). In total, four individuals had cerebral aneurysms leading to hemorrhages at ages 23–62, and two individuals died in their mid 40s after aortic dissection. There were two instances of autoimmune disorders (Crohn disease and Sjögren syndrome in family 1). Individual 2:II-1 had chronic hoarseness that resulted from an abnormality of the cricoarytenoid joint (A.V., unpublished data).

### Clinical Laboratory Studies

Electron microscopy examination of skin reported in families 4 and 5 showed decreased collagen content, abnormal variation in collagen fibril diameter, and some abnormally shaped fibrils.^3^, ^4^ Similar abnormalities were observed in skin from three individuals from family 1 (Figures S1 and S2) as well as individuals 2:II-1 and 5:V-6.^4^ Biochemical analysis of collagen in cultured skin fibroblasts in family 1 did not show abnormalities in the production and secretion of type I, III, and V collagens. Results of collagen analyses in individuals 5:III-3 and 8:II-2 were reported as normal,^4^, ^22^ as were those in cells from the probands in families 2, 3, 12, 13, 14, and 16. Complement studies in family 1 (CH50 and circulating levels of C1s and C1r) showed no consistent alterations in classical pathway activation (data not shown).

## Discussion

Periodontal EDS is a distinct clinical entity that we have now shown to be caused by mono-allelic missense or in-frame insertion/deletion alterations in *C1R* or *C1S*, the genes that encode complement 1 subunits C1r and C1s. The cardinal clinical feature is severe early-onset periodontitis with marked gingival recessions that in some individuals affects primary teeth. In contrast to individuals with non-syndromic chronic or aggressive periodontitis, those with pEDS have strikingly thin and fragile oral soft tissue with absence of attached gingiva (Figure 2). This feature of pEDS facilitates the clinical diagnosis prior to evident periodontitis: usually, the free gingival margin (the terminal edge of the gingiva surrounding the teeth) is continuous with the attached gingiva, which is tightly bound to the underlying periostum by collagenous anchoring fibrils that provide protection during chewing or tooth brushing (Figure 2). In pEDS-affected individuals, attached gingiva is lacking, and the thin and mobile alveolar mucosa directly proceeds to the free gingival margin, causing oral tissue fragility. Connective tissue pathology in pEDS also includes atrophic pretibial skin with areas of hyperpigmentation (83%), easy bruising (96%), and increased risk of arterial aneurysms (16%). Joint symptoms are generally mild, with hypermobility mostly of small joints.

**Figure 3:**
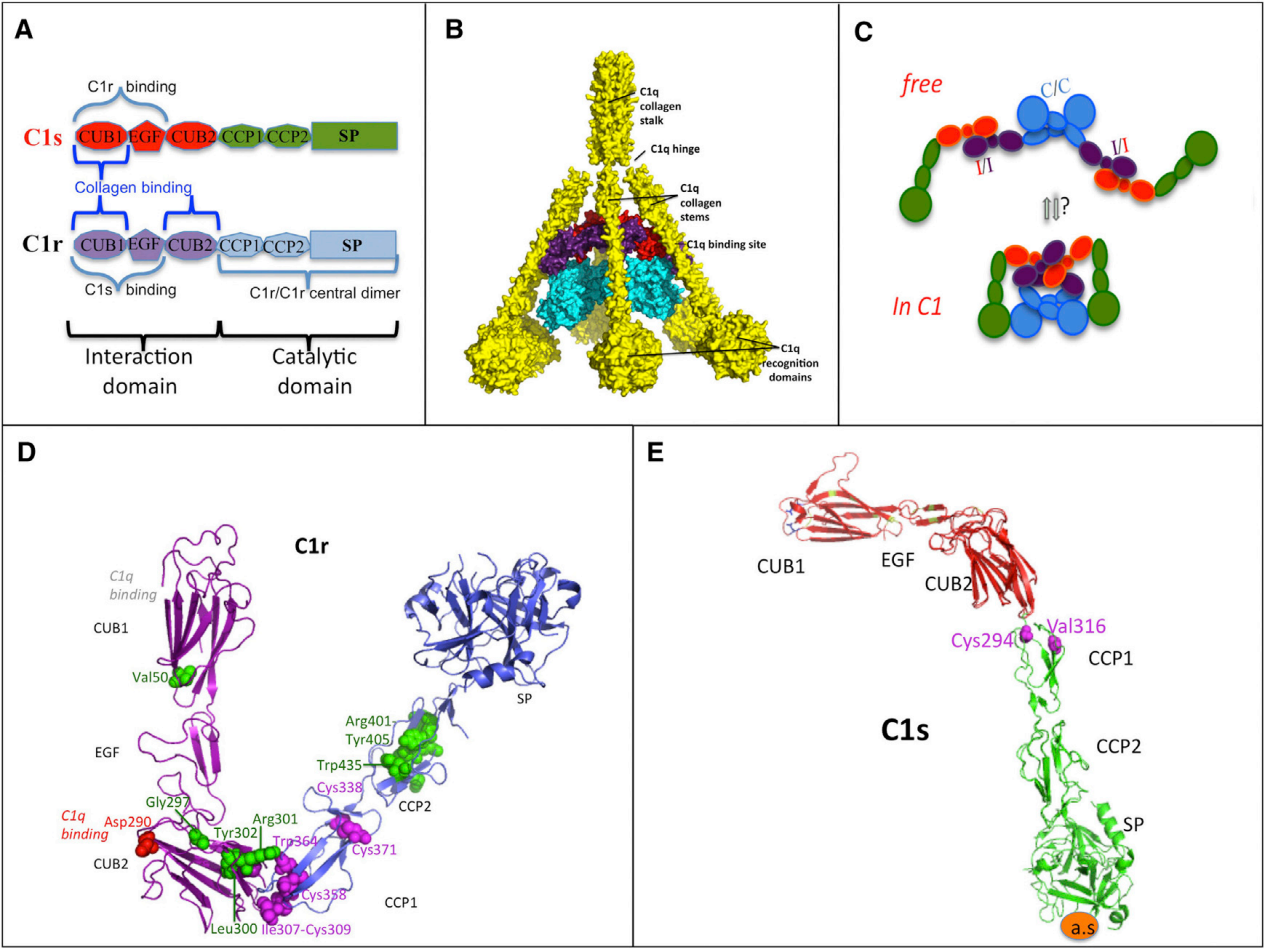
C1r and C1s Structure (A) Modular structure of C1r and C1s and main binding sites to assemble C1. The CUB domain (for complement C1r/C1s, uEGF, BMP1) is a structural motif of approximately 110 residues found almost exclusively in extracellular and plasma membrane-associated proteins. The EGF-like domain is an evolutionary conserved protein domain, which derives its name from the epidermal growth factor where it was first described. It comprises about 40 amino acid residues with six cysteines that form characteristic intra-domain disulfide bonds (1-3, 2-4, and 5-6). CCP (Complement Control Proteins) domains are also termed Sushi domains or Short Consensus Repeats and contain about 60 amino acid residues, each with 4 conserved cysteines that form intradomain disulfide bonds (1-3 and 2-4). These domains are involved in interaction between subunits of proteins and between proteins. The Serine Protease (SP) domains are mostly catalytic domains evolutionary related to the trypsin-chymotrypsin enzymes. The same color code is used for the domains throughout the figure. (B) C1q (yellow) is a hexamer of heterotrimers that contains in its cone the main protease interfacial domains that are crucial for C1r/C1s tetramer assembly. Each heterotrimer (A, B, C chains) contains a protease binding site in its collagen stem and a C-terminal globular recognition domain. This incomplete C1 model includes two copies each of C1r and C1s interaction domains (violet, red) and two copies of C1r catalytic domains (blue). (C) Schematic view of the main protease conformational changes during C1 assembly, with strong bending between the interaction and catalytic domains. The central C1r/C1r interface (C/C, blue) involves C1r CCP1 and SP head to tail interactions. (D and E) Mapping the C1r and C1s variants on 3D structure models. The wild-type residues affected by variants that cause pEDS are shown in colored spheres. The homologous modules are about the same size in the two proteases, which are shown at a different scale.

C1r and C1s are structurally similar proteins encoded by *C1R* and *C1S*, adjacent genes within the pEDS locus. Both proteins have an identical domain structure characterized by CUB1-EGF-CUB2-CCP1(Sushi)-CCP2(Sushi)-SP(serine protease) (Figure 3). Both proteins have amino-terminal signal sequences that direct them to the lumen of the rough endoplasmic reticulum. C1r and C1s associate as a proenzyme calcium-dependent tetramer that binds to a bouquet-like structure made of six C1q subunits to form the C1 complex. Each C1q subunit is a heterotrimer of A, B, and C chains that form a collagen-like stem (Figure 3). Upon binding of C1q to appropriate targets such as antigen-antibody complexes,^20^ C1r is auto-activated by cleavage at Arg463-Ile464 and can then cleave C1s at the parallel site (Arg447-Ile448) to form the active C1esterase. This enzyme can now cleave C4 and C2 to form the classical pathway C3 convertase (C4b2a).^23^, ^24^, ^25^

Heterozygous *C1R* or *C1S* mutations we identified in pEDS-affected individuals appear to have gain-of-function effects on as yet unidentified targets either within the cells or in the matrix. In contrast, complete deficiency of C1r or C1s caused by homozygous *C1R*- or *C1S*-null mutations causes a lupus-erythematosus-like syndrome with increased susceptibility to infections and increased risk of developing autoimmune diseases. Individuals heterozygous for *C1R*- or *C1S*-null mutations are reported to be asymptomatic, and in particular have not been reported to have periodontal disease.^13^, ^23^, ^26^ Loss of C1 esterase inhibitor results in intermittent and sometimes life-threatening angioedema due to excessive bradykinin production linked to an off-target effect of activated C1s.^27^ This is not a feature of pEDS.

Most mutations in our study alter residues that cluster at the hinges between the CUB2 and CCP1 modules, i.e., the interaction and catalytic domains of C1r and C1s (Figure 3). These hinges are the sites of a conformational change that allows the extended tetramer to fit into the C1q “cone.” Several mutations affect cysteines at positions 309/358 and 338/371 of C1r that form two stabilizing intra-chain disulfide bonds close to the C1r/C1r interface, which are essential for tetramer assembly (Figures 3A and 3C) and stabilization of Sushi modules (complement control protein [CCP] domains in complement and adhesion proteins). Disulfide bond formation could be indirectly affected by other identified mutations such as the C1R deletion-insertion mutation that involves residues 401–405 adjacent to the 406–447 disulfide bond or mutations that introduce additional cysteines. The C1R mutation c.869A>G (p.Asp290Gly, family 2) involves a C1q binding site and may interfere with the assembly of the C1 complex, as previously shown for p.Asp290Ala.^15^ The C1r p.Val50Asp substitution may affect the calcium-dependent interaction of C1r with C1s and consequently the interaction of the C1s-C1r-C1r-C1s tetramer with C1q.

**Figure 4:**
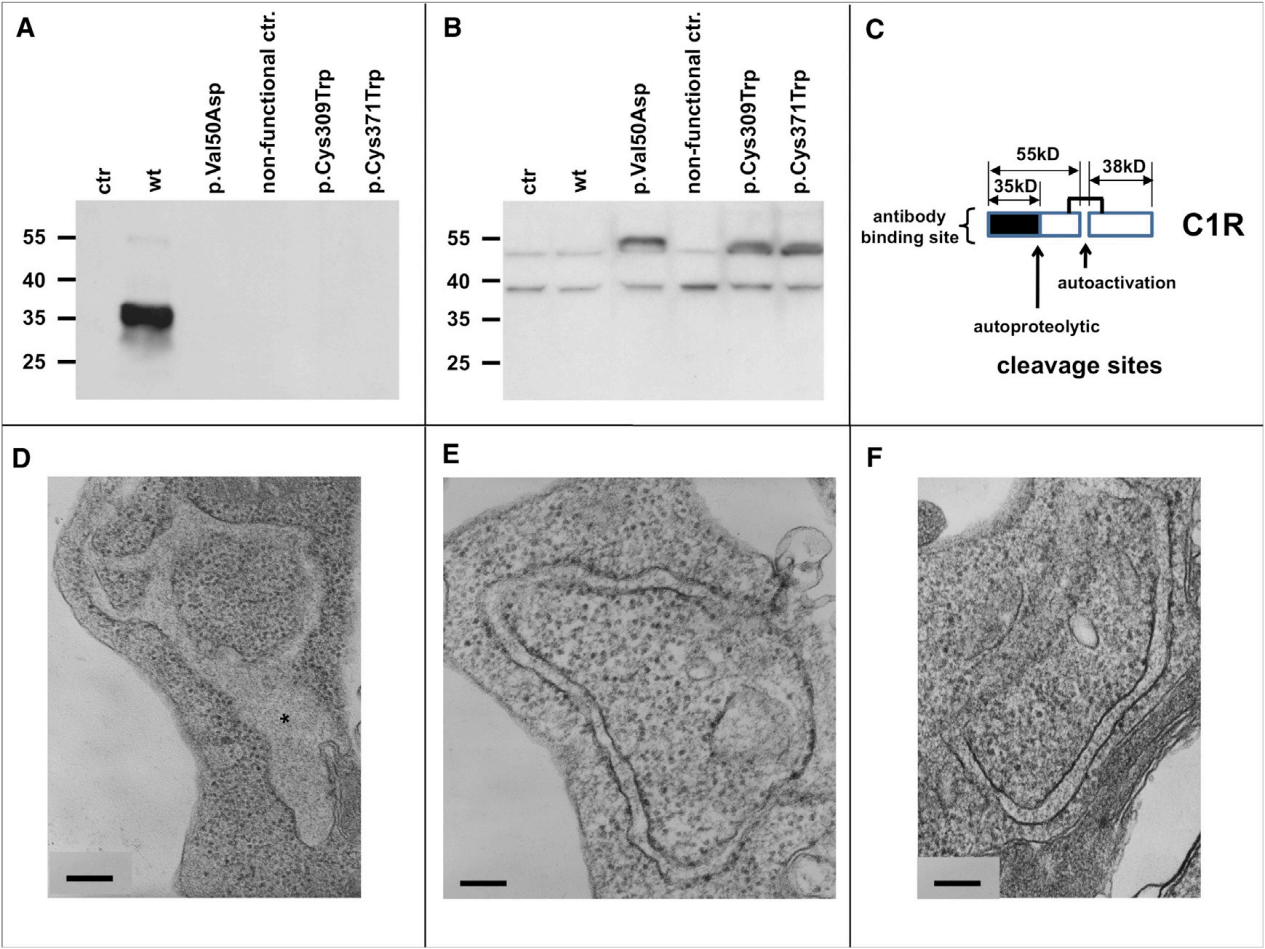
Analyses of Cells, Lysates, and Serum-free Supernatants of Transfected and Control HEK Cells (A) Western blot analysis of serum-free supernatants under reducing conditions (n = 3). The strong signal at 35 kDa in supernatant of wild-type *C1R* transfected HEK239 (WT) corresponds to the α-fragment of autocatalytically cleaved C1r A chain. Cell lines with missense mutations c.149_150TC>AT (p.Val50Asp), c.927C>G (p.Cys309Trp), and c.1113C>G (p.Cys371Trp), non-transfected controls (ctr), and a 26 bp frameshift insertion at position c.899_900 causing a nonsense mutation that is not expected to lead to a functional protein (nonfunctional ctr.) showed no extracellular signal for C1r. Coomassie staining was used as loading control. (B) Western blot analysis of cell lysates under reducing conditions (n = 3). The analysis of cell lysates in cell lines that express missense variants p.Val50Asp, p.Cys309Trp, and p.Cys371Trp showed additional bands at approx. 55 kDa corresponding to the uncleaved A chain of C1r. These bands are absent in non-transfected controls (ctr), transfected nonsense mutations (nonfunctional ctr), and wild-type (WT) C1r samples. This suggests a possible retention of mutated C1r within the cells. Loading control using α-tubulin antibody shows similar amounts of protein in all samples. (C) Schematic representation of C1r peptide subunits. C1r is autoactivated by cleavage into the 55 kDa A chain containing the domains CUB1-EGF-CUB2-CCP1-CCP2 and includes the binding domain, and the 28 kDa B chain which represents the serine protease domain; after activation the A chain is autoproteolytically cleaved into several fragments including a 35 kDa α-fragment (CUB1-EGF). The antibody used (Abcam cat# ab66751; RRID: AB_1860204) recognizes an N-terminal fragment encompassing residues 1–100 of human C1r (A chain). (D–F) Transmission electron microscopy. Ultrastructure of rough endoplasmic reticulum from mutation-transfected (D), wild-type-transfected (E), and untreated (F) HEK293 cells. Semiquantitative analysis of randomly selected section profiles showed RER dilatation (asterisk) in 36/63 profiles in cells transfected with c.1113C>G (p.Cys371Trp) compared to 18/60 profiles in cells transfected with the wild-type sequence, and 13/38 profiles in non-transfected cells. Scale bar represents 200 nm in all panels.

In order to study the effects of mutations observed in patients with pEDS, we overexpressed C1r variants p.Val50Asp, p.Cys309Trp, and p.Cys371Trp in HEK293 cells. Western blots of cells and supernatants indicated that the abnormal C1r proteins are retained in the cells but can undergo autoactivation that may lead to interaction with off-target substrates. Mutation-transfected cells showed an increased proportion of dilated RER cisternae (Figure 4), similar to that seen in skin in situ (provided in the Supplemental Data). The C1r-C1s tetramer normally binds to the N-terminal collagenous domain of C1q that contains a phylogenetically conserved hexapeptide motif Hyp-Gly-Lys-(Val/Asn)-Gly-(Pro//Lys/Met).^28^, ^29^ Hyp-Gly-Lys-Asn-Gly sequences are present in the triple helical domains of the proα1(I) and proα2(I) chains of type I collagen, as well as the proα1(III) chains of type III collagen, and may represent alternative C1r/C1s binding sites. C1q binding of C1r and C1s is mediated by the CUB domains that are evolutionarily conserved and are present in a number of proteins including procollagen C-proteinase enhancers (PCPE1) and bone-morphogenetic protein (BMP1).^30^ Both PCPE1 and BMP1, as well as C1s, can bind through their CUB domains to the triple helix of collagen and/or propeptides that can be degraded.^31^, ^32^, ^33^, ^34^, ^35^ This suggests that abnormal binding of (mutated) C1r/C1s to connective tissue precursors could be a pathogenetic factor in pEDS.

There is substantial evidence that altered complement function plays an important role in the pathogenesis of non-syndromic periodontitis.^36^ Induction of experimental gingivitis in human volunteers causes progressive complement activation (as determined by C3 conversion in gingival crevicular fluid) that is correlated with increased clinical inflammation.^37^ Conversely, traditional periodontal treatment can lead to decreased complement activity^38^ and C3 downregulation.^39^ Local inhibition of C3 reduced experimental periodontitis in non-human primates, and this strategy has been suggested as a treatment in humans.^40^ No mutations in *C1R* or *C1S* were detected in 71 individuals with aggressive periodontitis, which is a main differential diagnosis to pEDS. Aggressive periodontitis is a rare (prevalence 0.1% to 0.5%) complex genetic disease with familial aggregation, characterized by rapid progressing periodontal destruction in otherwise healthy individuals, typically occurring before the age of 35 years. Also, no mutations in *C1R* or *C1S* were detected in families 18 and 19. Individuals with suggested pEDS in these families presented with periodontitis and EDS-type connective tissue features but had no pretibial plaques.^5^, ^19^ The proposita in family 19 had only moderate periodontal destruction at age 37 (community periodontal index of treatment needs [CPITN] grade III) and a history of severe caries, suggesting chronic periodontitis.^19^ Neither family was available for clinical re-assessment.

In conclusion, pEDS in at least the great majority of cases results from specific classes of heterozygous mutations in *C1R* and *C1S*. The mechanism of pathogenesis of these mutations differs from homozygous loss of function of these genes and from loss of the C1 esterase inhibitor. Clinical diagnosis of pEDS should be based on severe periodontitis with early onset in combination with absence of attached gingiva, as well as pretibial hyperpigmentation and easy bruising and confirmation by genetic tests. Individuals should receive specific surveillance for aneurysms.

## Consortia

Additional members of the Molecular Basis of Periodontal EDS Consortium are Kirk Aleck, Zoltan Banki, Joszef Dudas, Herbert Dumfahrt, Hady Haririan, James K. Hartsfield, Charles N. Kagen, Uschi Lindert, Thomas Meitinger, Wilfried Posch, Christian Pritz, David Ross, Richard J. Schroer, Georg Wick, Robert Wildin, and Doris Wilflingseder.

## Figures and Tables

**Table 1:** Identified Pathogenic Variants in the Present Cohort with pEDS

**Family**	**Affected (n)**	**Gene**	**DNA (c.) (GRCh38)**	**Protein, p. (Mature Protein)**	**Domain**
1	15	*C1R*	c.149_150TC>AT	p.Val50Asp (Val32Asp)	CUB1 (EGF)
2	1	*C1R*	c.869A>G	p.Asp290Gly (Asp272Gly)	C1q binding site
3	1	*C1R*	c.890G>A	p.Gly297Asp (Gly279Asp)	CUB2
4	3	*C1R*	c.899T>C	p.Leu300Pro (Leu282Pro)	CUB2 (near CCP1)
5	13	*C1R*	c.902G>C	p.Arg301Pro (Arg283Pro)	CUB2 (near CCP1)
6	7	*C1R*	c.905A>G	p.Tyr302Cys (Tyr284Cys)	CUB2 (near CCP1)
7	1	*C1R*	c.917_927delinsGGACA	p.Ile306_Cys309del-insArgArg (Ile288_Cys291 del-insArgArg	Sushi CCP1
8	1	*C1R*	c.927C>G	p.Cys309Trp (Cys291Trp)	Sushi CCP1 (near CUB2)
9	3	*C1R*	c.927C>G	p.Cys309Trp (Cys291Trp)	Sushi CCP1 (near CUB2)
10	4	*C1R*	c.1012T>C	p.Cys338Arg (Cys320Arg)	Sushi CCP1 (near CCP2)
11	3	*C1R*	c.1073G>T	p.Cys358Phe (Cys340Phe)	Sushi CCP1 (near CUB2)
12	1	*C1R*	c.1092G>C	p.Trp364Cys (Trp346Cys)	Sushi CCP1 (near CUB2)
13	1	*C1R*	c.1113C>G	p.Cys371Trp (Cys353Trp)	Sushi CCP1 (near CCP2)
14	10	*C1R*	c.1200_1215delinsTCATGTAATA	p.Arg401_Tyr405del-insHisValIle (Arg383_Tyr387del-insHisValIle)	Sushi CCP2
15	12	*C1R*	c.1303T>C	p.Trp435Arg (Trp417Arg)	Sushi CCP2
16	7	*C1S*	c.880T>C	p.Cys294Arg (Cys279Arg)	Sushi CCP1 (near CUB2)
17	9	*C1S*	c.945-947del	p.Val316del (Val301del)	Sushi CCP1 (near CUB2)

Abbreviations are as follows: n, number; C1R, complement 1 subcomponent r; C1S, complement 1 subcomponent s. For both *C1R* and *C1S*, c.1 is the first nucleotide of the initiator codon and p.1 is the initiator methionyl residue. The GenBank reference sequences used are NM_001733 and NM_001734 for *C1R* and *C1S*, respectively. The signal sequences for C1r and C1s are 18 and 15 amino acids in length, respectively. Most of the literature about these proteins uses p.Ser19 (C1r) and p.Glu16 (C1s) for the start residues of these two proteins. We have included the reference in the mature protein alignment for the sites of the pathogenic variant in parentheses.

**Table 2:** Summary of Clinical Features in Periodontal EDS

**Clinical Features**	**Prevalence**
**Oral Features**	

Early-onset periodontitis^a^	99%
Gingival recessions	98%
Thin gingiva and/or absence of attached gingival	93%

**Skin**	

Easy bruising	96%
Pretibial hyperpigmentation (not observed in family 1)	83%
Skin fragility	83%
(Mild) elastic skin	73%
Abnormal scarring (atrophic or wide)	50%
Prominent vasculature	50%

**Joint Features**	

Joint hypermobility^b^	44%
Joint pain	31%
Flat feet	30%
Scoliosis	22%
Osteoarthritis	9%
Joint dislocation	4.8%

**Others**	

Recurrent infections (e.g., bladder, epididymitis, eye, zoster, otitis media)	40%
Marfanoid facial features	30%
Hernia (inguinal, umbilical, hiatal, abdominal, surgical)	25%
Aneurysms (present only in families 5, 6, and 14)	16%
Cancer (more prevalent in individuals with *C1S* mutations)	11%
Autoimmune disorder (present only in family 1)	7.7%
Organ rupture (3 times in individual 1:III-10)	—

Prevalence rates are based on 93 individuals with mutations in *C1R* or *C1S*, and with respective clinical data from the present cohort (Table S1).

a Age of first tooth loss, 2–30 years; age of complete tooth loss, 14–48 years; prepubertal periodontitis (age <10 years), 16%.

b Fingers, 30%; elbows, 19%; knees, 11%; hips, wrist, and ankle, 3%.

## Accession Numbers

All variants have been submitted to ClinVar (submission numbers SUB1882099, SUB1882115, SUB1882116, and SUB1882151).

## Web Resources

1000 Genomes, http://www.1000genomes.org
ClinVar, https://www.ncbi.nlm.nih.gov/clinvar/
dbSNP, http://www.ncbi.nlm.nih.gov/projects/SNP/
ExAC Browser, http://exac.broadinstitute.org/
GenBank, http://www.ncbi.nlm.nih.gov/genbank/
OMIM, http://www.omim.org/
RCSB Protein Data Bank, http://www.rcsb.org/pdb/home/home.do
RRID, https://scicrunch.org/resources

## Supplemental Data

Document S1. Descriptions of Families and Figures S1 and S2

Table S1. Clinical Features of Each Individual with pEDS

Document S2. Article plus Supplemental Data
