# Supplementary material for: Periodontal Ehlers-Danlos Syndrome Is Caused by Mutations in C1R and C1S, which Encode Subcomponents C1r and C1s of Complement
Source: Am J Hum Genet. 2016 Oct 13;99(5):1005–14. doi: 10.1016/j.ajhg.2016.08.019 (PMC5097948; doi:10.1016/j.ajhg.2016.08.019)
Supplement: Document S1. Descriptions of Families and Figures S1 and S2 [file mmc1.pdf]

The American Journal of Human Genetics, Volume *99*

Supplemental Data

Periodontal Ehlers-Danlos Syndrome Is Caused by Mutations in *C1R* and *C1S*, which Encode Subcomponents C1r and C1s of Complement

Ines Kapferer-Seebacher, Melanie Pepin, Roland Werner, Timothy J. Aitman, Ann Nordgren, Heribert Stoiber, Nicole Thielens, Christine Gaboriaud, Albert Amberger, Anna Schossig, Robert Gruber, Cecilia Giunta, Michael Bamshad, Erik Björck, Christina Chen, David Chitayat, Michael Dorschner, Marcus Schmitt-Egenolf, Christopher J. Hale, David Hanna, Hans Christian Hennies, Irene Heiss-Kisielewsky, Anna Lindstrand, Pernilla Lundberg, Anna L. Mitchell, Deborah A. Nickerson, Eyal Reinstein, Marianne Rohrbach, Nikolaus Romani, Matthias Schmuth, Rachel Silver, Fulya Taylan, Anthony Vandersteen, Jana Vandrovcova, Ruwan Weerakkody, Margaret Yang, F. Michael Pope, Molecular Basis of Periodontal EDS Consortium, Peter H. Byers, and Johannes Zschocke

Supplemental Case Reports: Clinical description of families with periodontal EDS

Detailed clinical data on each individual with periodontal EDS are provided in the Supplemental Table 1.

Family 1, *C1R* c.149_150TC>AT, p.Val50Asp (n = 15):

Subject 1:IV-1 presented at age 21 to dental clinic seeking treatment for aggressive periodontitis (EOP) (age of onset 11 years). The identified *C1R* mutation segregated with fifteen affected individuals in this 5-generation Austrian family. EOP in eleven individuals led to early tooth loss in the teens or early twenties. Three individuals were children aged five to nine years, not yet affected by EOP, but with extreme gingival inflammation despite good oral hygiene and professional tooth cleaning on a regular basis. One affected adult subject (24 years old) presented without EOP despite heavy deposits, but with severe gum fragility and extensive gingival recessions, as well as mild joint hypermobility of the digits and the elbows. Intraoral examination in 15 affected family members revealed a structural soft tissue defect characterized by a lack of attached gingiva (Figure 2 in the main article). Joint hypermobility of fingers and elbows was noted on exam in 56% of affected family members. Dermatological findings included easy bruising (77%) and soft, dry skin (55%). Unlike previous reported families with periodontal EDS, no affected individuals demonstrated pretibial discoloration. Three family members reported a history of autoimmune disorders including Crohn's disease (1:III-10), Sjögren's syndrome (1:III-2), and rosacea (1:IV-1). One individual (1:III-10) had a history of recurrent pneumothoraces, an intestinal perforation, and inguinal hernia. On child (1:V-8; 9 years) had recurrent Herpes zoster.

Collagen biochemical analysis in individuals 1:III-10, 1:IV-10, and 1:IV-2 showed normal relative proportions of collagens I:III:V, as well as normal electrophoretic mobility and no over modification or intracellular retention of α1(I) and α2(I) collagens (including β chains), $\alpha 1(III)_3$ collagen trimers, and α1(V) and α2(V) collagens.

Family 2 *C1R* c.869A>G, p.Asp290Gly (n = 1)(separately reported by George et al. in press[1]):

The proband (3:II-1) was a 33-year-old Caucasian female with premature loss of her primary teeth, loss of numerous adult teeth by age 19, and pretibial bruising since early childhood. During her twenties, she developed a hoarse voice. She has mild distal hypermobility of her metacarpophalangeal joints, broad feet and shortening of her 5th metatarsal bilaterally. Her father had EOP.

Family 3, *C1R* c.890G>A, p.Gly297Asp (n = 1):

The proband was a 56-year old Caucasian woman. EOP had been diagnosed at age 10, when the first permanent teeth were lost. Despite periodontal treatment, complete tooth loss occurred at age 43. She reported on generalized gum thinning. There was no joint hypermobility or other joint features. Hyperextensibility of the skin was first diagnosed at age 22; there are marked pretibial discolorations, prominent vasculature, and thickened scars. She reported on recurrent wound infections with prolonged healing, and frequent kidney infections.

Family 4 *C1R* c.899T>C, p.Leu300Pro (n = 3)(previously reported by Reinstein et al. 2013[2]):

Four individuals in four generations were described with EOP, tooth loss in the teens and early twenties, and pretibial discoloration in three (4:II-1, 4:III-1, and 4:IV-1). The gingival soft tissues were minimally attached with keratinized gingivae. Skin was of normal consistency, with no hyperextensibility, and scar formation was normal. The individual (4:I-1), described with dental features of periodontal EDS but absent skin and joint findings was not confirmed to have the familial *C1R* mutation, evidence that either the mutation was de-novo in individual (4:II-1) or mosaic in individual (4:I-1).

Family 5 *C1R* c.902G>C, p.Arg301Pro (n = 13)(previously reported by Rahman et al. 2003[3]):

Seventy-two individuals from a Swedish five-generation family were examined previously. The predominant dental features in the affected indviduals (n = 17) were premature periodontal inflammation and gingival recession, usually detectable in childhood, with rapid progression throughout adolescence and early adulthood. In general, complete loss of adult dentition occurred typically by the end of the third decade. Dental X-rays of selected family members showed progressive loss of periodontal supporting tissue and loss of bony tissue. Affected individuals showed typical features of EDS, with generalized joint laxity (Beighton scores 5/9–9/9), thin atrophic skin (especially over the dorsum of the hands and feet), and circumscribed hemosiderotic pretibial plaques, which could be thickened or atrophic. Affected individuals were tall, with spans wider than their heights.

Family 6 *C1R* c.905A>G, p.Trp302Cys (n = 7): The proband (6:III-1) presented at 26 years of age to medical genetics to be evaluated for EDS type IV because of a family history of early death from arterial rupture in four maternal relatives, a personal history of skin fragility, easy bruising, hyperelastic skin and loose teeth leading to elective dentures at age 21 years. Two daughters (6:IV-2 and 6:IV-3) presented with receding gums and periodontitis before age 10. Pretibial discoloration, EOP and gingival recessions were noted in the proband and in the four relatives with aneurysm. The probands mother (6:II-2) died at age 46 after aortic dissection, the

proband's maternal uncle (6:II-3) died at age 23 and the proband's maternal aunt (6:II-4) died at age 42 after haemorrhage of a cerebral aneurysm, and the proband's maternal grandfather died at age 43 after heart attack.

Family 7 *C1R* c.917_927delinsGGACA, p.Ile306_Cys309delinsArgArg (n = 1)(previously reported by Cikla et al. 2014[4]):

The proband 7 II-1was initially evaluated at age 13 years. She presented with frequent bruising, fragility of the skin over the anterior aspects of her tibiae (over one of which she experienced an avulsion laceration), and periodontal bone loss with gingival recession. All of her teeth had either been lost spontaneously or had been extracted by the time she was 19 years old. The areas over her tibiae episodically swelled for prolonged periods, following which there was resolution into persistent hyperpigmented lesions. She had mild joint hyperextensibility but no hyperextensible skin. At age 42, she experienced a subarachnoid hemorrhage with an intraventricular and intraparenchymal hemorrhage due to an aneurysm of the left middle cerebral artery, resulting in a poor neurologic condition after surgery.

Family 8 *C1R* c.927C>G, p.Cys309Trp (n = 1)(previously reported by Hartsfield and Kousseff 1990[5]):

The female proband (7:II-2) presented at 12 years of age for evaluation for EDS. Periodontal EDS was clinically diagnosed based on EOP with tooth loss at age 17, increased skin fragility with minimal stretchability, atrophic scars, mild joint laxity mostly in the digits, kyphoscoliosis, easy bruising, and pretibial ecchymotic lesions. The gingiva was described as friable and edematous, with bleeding after tooth brushing and minor trauma since age 2 years. She had no family history of similar medical problems.

Family 9 *C1R* c.927C>G, p.Cys309Trp (n = 3):

The male proband (8:II-1) was evaluated at 35 years of age by medical genetics for features of EDS including easy bruising, hyperelastic skin, pretibial discoloration, poor wound healing and EOP. His two daughters were identified to have early periodontitis and gum recession before age 10.

Family 10 *C1R* c.1012T>C, p.Cys338Arg (n = 4):

At 48 years of age, female proband (9:I-1) was referred to medical genetics for evaluation of EDS with distal joint hypermobility, thin stretchy skin, atrophic scars, skin fragility, easy bruising,

and pretibial discoloration in addition to EOP with gingival recessions and gum fragility. Her affected daughter and two granddaughters share all noted features.

Family 11 *C1R* c.1073G>T, p.Cys358Phe (n = 3)(previously reported by Stewart et al 1977[6]):

The male proband (10:II-2) presented at 21 years of age to medical genetics with a primary complaint of loose teeth as a result of EOP, fragile skin, particularly in the pretibial area of his legs, tall stature, arachnodactyly, hypermobility of fingers and peculiar scars. His father and half-brother were similarly affected.

Family 12 *C1R* c.1092G>C, p.Trp364Cys (n = 1):

A 3 year old Caucasian male (11:II-2) was referred to medical genetics for evaluation of possible EDS, with easy bruising, fragile skin, pretibial discoloration and thin hair. He had no family history of similar findings. Follow-up is unavailable.

Family 13 *C1R* c.1113C>G, p.Cys371Trp (n = 1): A 6 year old Caucasian male (12:II-2) was referred to medical genetics for evaluation of possible EDS, joint laxity, tibial bruising, gum bleeding and gingival recession. On exam he was identified to have hypermobility of fingers, elbows and wrists, fragile skin, easy bruising, pretibial discoloration and EOP. He had no family history of similar findings

Family 14 *C1R* c.1200_1215delinsTCATGTAATA, p.Arg401_Tyr405delinsHisValIle (n = 10):

The female proband (13:III-4) was referred to medical genetics at 30 years of age to be evaluated for EDS with easy bruising, hyperelastic and fragile skin resulting in pretibial discoloration, gingival recession, EOP and tooth loss as a result of loose teeth beginning in her teens. Each relative confirmed to have the *C1R* mutation has features of periodontal EDS consistently including gum recession, EOP and tooth loss at a young age.

Family 15 *C1R* c.1303T>A, p.Trp435Arg (n = 12):

In this Swedish family, twelve individuals in five generations have been described with gingival inflammation and ulcerations since early childhood, and periodontitis in the childhood or teens. In subject 15:IV-4, periodontal bone loss was diagnosed radiographically at nine years of age. Lack of attached gingiva was diagnosed in several affected individuals. Pretibial discolorations were present in nine individuals, and were associated with swollen legs and painful varices in

individual 15:III-5. In individual 15:I-2, back of hands had discolorations. Two individuals suffered from frequent joint subluxations and joint pain. Hypermobility of thumbs, fingers, ribs and toes was present in two individuals. In this family, there is also a history of bleeding tendency (placental bleeding during pregnancy in 15:III-5, and bleeding after surgery in 15:II-3), and of recurrent infections. One individual died at eight years of age due to a nasal connective tissue tumor. Another individual (15:I-1) had a cerebral aneurysm leading to hemorrhages at ages 40 and 62. One individual (15:II-2) who is not a mutation carrier has hypermobility, subluxations and suffered from a profuse bleeding after hysterectomy.

Family 16 *C1S* c.880T>C, p.Cys294Arg (n = 7):

The female proband (14:II-2) presented to medical genetics at 45 years of age for evaluation of EDS with mild elastic skin, easy bruising, fragile skin with pretibial discoloration and EOP leading to tooth loss in her teens. A similar phenotype was described in her father, sister, brother and sons. In those confirmed to harbor the *C1S* mutation, clinical heterogeneity was noted. The extent of pretibial discoloration varied from near absent to extensive and the age of onset of EOP from the first decade to the third. As adults II-2 and II-3 suffered significant spinal osteoarthritis, fragile skin on legs and fingers, irritable bowel and flu-like symptoms without fever. Three affected relatives were diagnosed with carcinoma.

Family 17 *C1S* c.945-947del, p.Val316del (n = 9):

The male proband (15:III-1) presented to medical genetics at 75 years of age with a personal and family history of EOP and features of periodontal EDS including easy bruising, thin, fragile hyperelastic skin, atrophic scars, pretibial discoloration and scoliosis. Periodontitis was initially noted in his 20's, with first tooth loss at 26 years of age and loss of 20 teeth by age 60. At 63 he had a successful dental implant. Eight out of nine affected relatives shared EOP, gingival recession, early tooth loss and pretibial discoloration. There was a family history of neoplasm in four affected relatives including uterine (15:II-1), breast (15:II-3), lymphocytic lymphoma of the lung (15:III-1) and colon (15:III-3).

Family 18, no mutation in *C1R* or *C1S* (n = 12)(previously reported by Reinstein et al. 2011[7]):

This US family with twelve affected individuals in four generations has been followed over 12 years. The phenotype in this family is characterized by joint hypermobility (10 out of 12), joint dislocations (8 out of 12), normal scar formation but eventual scar atrophy, and severe periodontal disease (10 out of 12) with dental caries and infections. Ostheoarthritis and scoliosis were reported in 5 out of 12 individuals. The age of onset and severity of symptoms was quite variable amongst the affected individuals. No one in this family had the pretibial

scarring and discoloration, In addition, the presence of a marfanoid habitus was not observed in any of the probands described.

Family 19, no mutation in *C1R* or *C1S* (n = 3)(previously reported by Reinstein et al. 2012[8]):

The patient was a 37-year-old woman with moderate periodontitis (class III; with IV being the most severe according to the American Dental Association classification). She reported on early tooth extractions before age 18 months because of black and rotten teeth, suggesting a cariological problem rather than periodontal disease. The patient reported being very flexible and double jointed from childhood but did not dislocate any joints. During adolescence she developed scoliosis. She first experienced lower back, hip and knee joint pain in her early 20's. The patient is now experiencing chronic pain in her back, knees, hips and most of her finger joints and is treated with high dose of opiate analgesics. Other relevant medical history includes easy bruising with normal healing and normal scar formation. Her mother and maternal grandmother have the same medical condition.

Supplemental figures

Figure S1: Ultrastructural investigations of the reticular dermis from two affected individuals from family 1.

(A and B) Transmission electron microscopy of the skin biopsy of individual 1:III-10 shows abnormally enlarged endoplasmic reticulum cisterns (arrows), and collagen fibrils of variable diameter, size and slightly irregular contours. **(C and D)** Transmission electron microscopy of the skin biopsy of 1:IV-10 shows similar results. Note the absence of cauliflower-like fibrils and the presence of microfibrillar meshes between the collagen fibrils. For each image, scale bars are indicated.

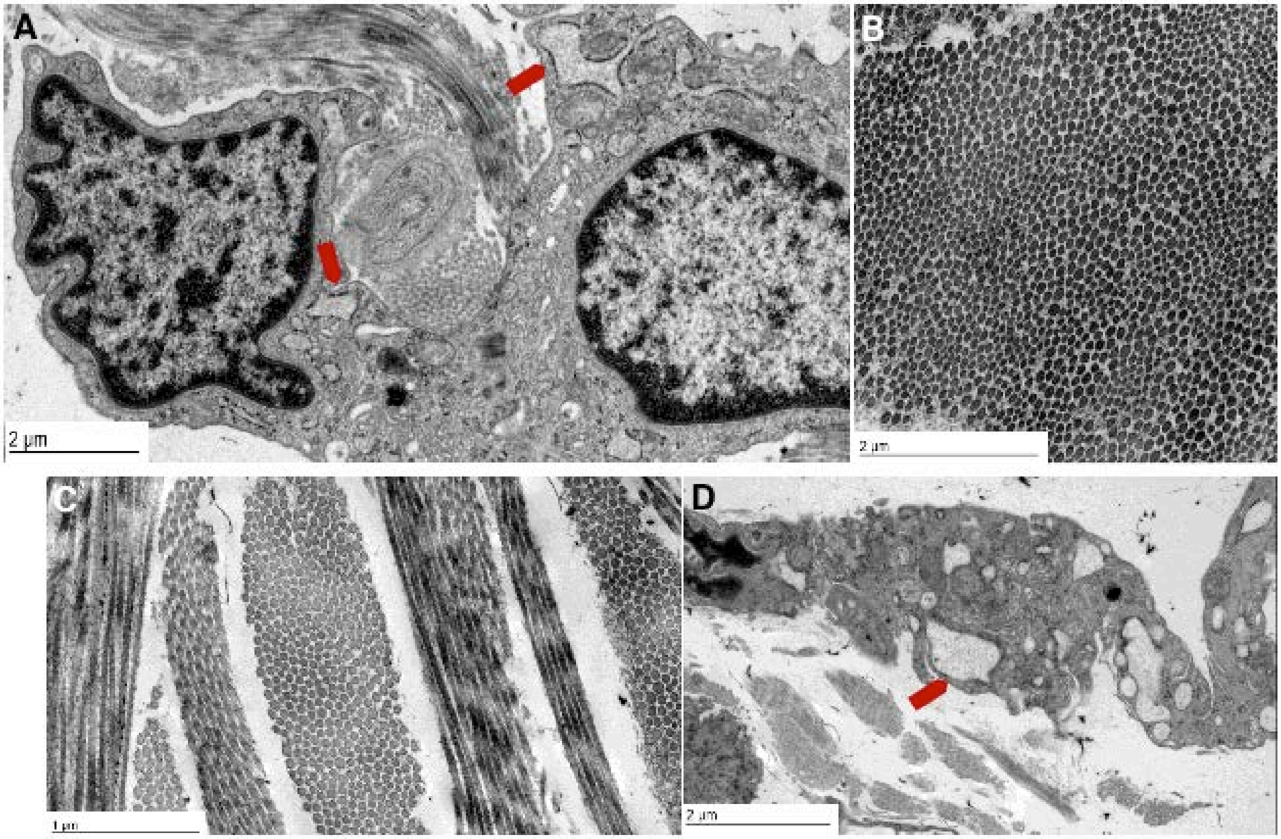

Figure S2. **Ultrastructure of cultured fibroblasts**

Transmission electron microscopy of fibroblast from patients 1:III-10 **(A)**, 1:IV-10 **(B)** and 1:IV-2 **(C)** shows the enlargement of the rough endoplasmic reticulum cisterns compared to control fibroblasts **(D)**. Scale bars are reported.

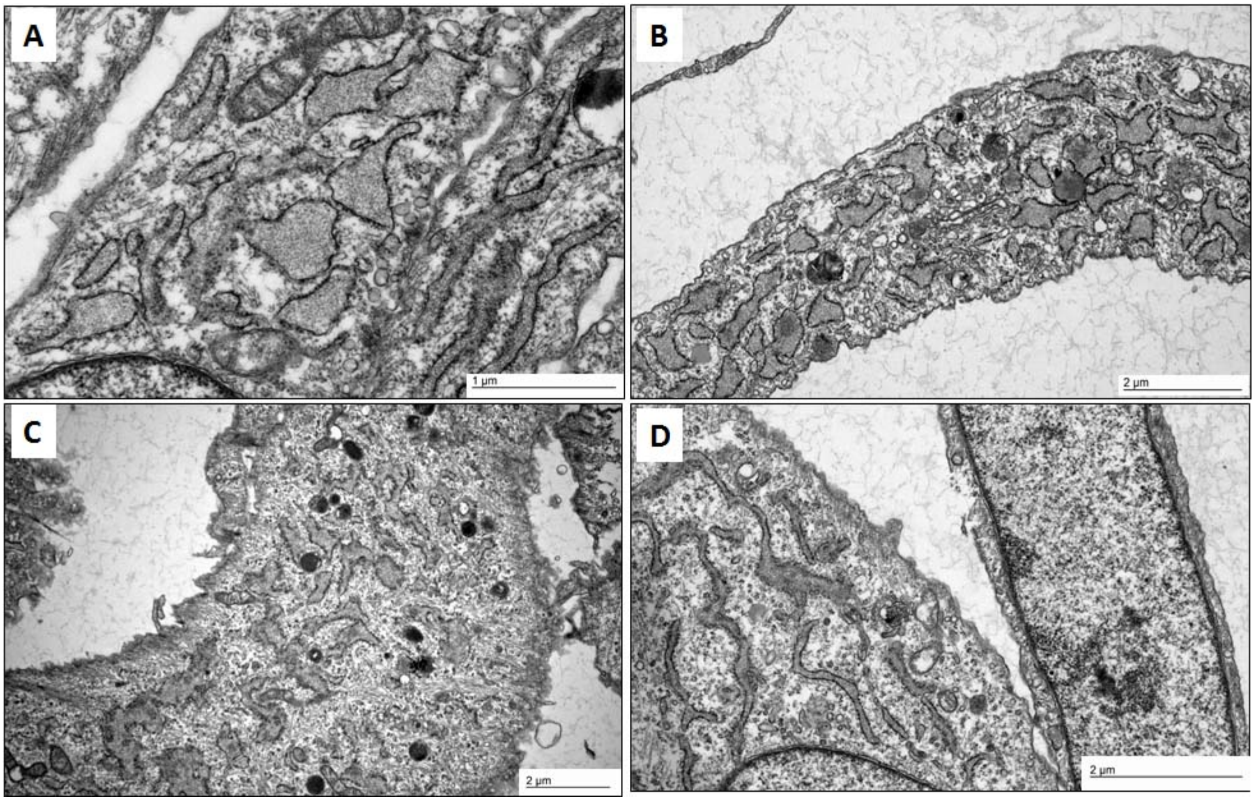

Supplemental References

1. George, S.M.C., Vandersteen, A., Nigar, E., Ferguson, D.J.P., Topham, E.J., and Pope, F.M. (in press). Two Ehlers Danlos syndrome type VIII patients with unexpected hoarseness. Clinical and Experimental Dermatology.
2. Reinstein, E., DeLozier, C.D., Simon, Z., Bannykh, S., Rimoin, D.L., and Curry, C.J. (2013). Ehlers-Danlos syndrome type VIII is clinically heterogeneous disorder associated primarily with periodontal disease, and variable connective tissue features. European journal of human genetics : EJHG 21, 233-236.
3. Rahman, N., Dunstan, M., Teare, M.D., Hanks, S., Douglas, J., Coleman, K., Bottomly, W.E., Campbell, M.E., Berglund, B., Nordenskjold, M., et al. (2003). Ehlers-Danlos syndrome with severe early-onset periodontal disease (EDS-VIII) is a distinct, heterogeneous disorder with one predisposition gene at chromosome 12p13. American journal of human genetics 73, 198-204.
4. Cikla, U., Sadighi, A., Bauer, A., and Baskaya, M.K. (2014). Fatal Ruptured Blood Blister-like Aneurysm of Middle Cerebral Artery Associated with Ehlers-Danlos Syndrome Type VIII (Periodontitis Type). J Neurol Surg Rep 75, e210-213.
5. Hartsfield, J.K., Jr., and Kousseff, B.G. (1990). Phenotypic overlap of Ehlers-Danlos syndrome types IV and VIII. American journal of medical genetics 37, 465-470.
6. Stewart, R.E., Hollister, D.W., and Rimoin, D.L. (1977). A new variant of Ehlers-Danlos syndrome: an autosomal dominant disorder of fragile skin, abnormal scarring, and generalized periodontitis. Birth defects original article series 13, 85-93.
7. Reinstein, E., Wang, R.Y., Zhan, L., Rimoin, D.L., and Wilcox, W.R. (2011). Ehlers-Danlos type VIII, periodontitis-type: further delineation of the syndrome in a four-generation pedigree. American journal of medical genetics Part A 155A, 742-747.
8. Reinstein, E., Pariani, M., Lachman, R.S., Nemec, S., and Rimoin, D.L. (2012). Early-onset osteoarthritis in Ehlers-Danlos syndrome type VIII. American journal of medical genetics Part A 158A, 938-941.
